# Automated Digital Safety Planning Interventions for Young Adults: Qualitative Study Using Online Co-design Methods

**DOI:** 10.2196/69602

**Published:** 2025-02-26

**Authors:** Jonah Meyerhoff, Sarah A Popowski, Tanvi Lakhtakia, Emily Tack, Rachel Kornfield, Kaylee P Kruzan, Charles J Krause, Theresa Nguyen, Kevin Rushton, Anthony R Pisani, Madhu Reddy, Kimberly A Van Orden, David C Mohr

**Affiliations:** 1 Department of Preventive Medicine Feinberg School of Medicine Northwestern University Chicago, IL United States; 2 Mental Health America Alexandria, VA United States; 3 Department of Psychiatry School of Medicine and Dentistry University of Rochester Rochester, NY United States; 4 Department of Pediatrics School of Medicine and Dentistry University of Rochester Rochester, NY United States; 5 Department of Informatics Donald Bren School of Information and Computer Sciences University of California Irvine, CA United States

**Keywords:** mental health services, technology, therapy, computer assisted, SMS text messaging

## Abstract

**Background:**

Young adults in the United States are experiencing accelerating rates of suicidal thoughts and behaviors but have the lowest rates of formal mental health care. Digital suicide prevention interventions have the potential to increase access to suicide prevention care by circumventing attitudinal and structural barriers that prevent access to formal mental health care. These tools should be designed in collaboration with young adults who have lived experience of suicide-related thoughts and behaviors to optimize acceptability and use.

**Objective:**

This study aims to identify the needs, preferences, and features for an automated SMS text messaging–based safety planning service to support the self-management of suicide-related thoughts and behaviors among young adults.

**Methods:**

We enrolled 30 young adults (age 18-24 years) with recent suicide-related thoughts and behaviors to participate in asynchronous remote focus groups via an online private forum. Participants responded to researcher-posted prompts and were encouraged to reply to fellow participants—creating a threaded digital conversation. Researcher-posted prompts centered on participants’ experiences with suicide-related thought and behavior-related coping, safety planning, and technologies for suicide-related thought and behavior self-management. Focus group transcripts were analyzed using thematic analysis to extract key needs, preferences, and feature considerations for an automated SMS text messaging–based safety planning tool.

**Results:**

Young adult participants indicated that an automated digital SMS text message–based safety planning intervention must meet their needs in 2 ways. First, by empowering them to manage their symptoms on their own and support acquiring and using effective coping skills. Second, by leveraging young adults’ existing social connections. Young adult participants also shared 3 key technological needs of an automated intervention: (1) transparency about how the intervention functions, the kinds of actions it does and does not take, the limits of confidentiality, and the role of human oversight within the program; (2) strong privacy practices—data security around how content within the intervention and how private data created by the intervention would be maintained and used was extremely important to young adult participants given the sensitive nature of suicide-related data; and (3) usability, convenience, and accessibility were particularly important to participants—this includes having an approachable and engaging message tone, customizable message delivery options (eg, length, number, content focus), and straightforward menu navigation. Young adult participants also highlighted specific features that could support core coping skill acquisition (eg, self-tracking, coping skill idea generation, reminders).

**Conclusions:**

Engaging young adults in the design process of a digital suicide prevention tool revealed critical considerations that must be addressed if the tool is to effectively expand access to evidence-based care to reach young people at risk for suicide-related thoughts and behaviors. Specifically, automated digital safety planning interventions must support building skillfulness to cope effectively with suicidal crises, deepening interpersonal connections, system transparency, and data privacy.

## Introduction

### Background

The prevalence of suicidal thoughts among young adults, those between the ages of 18 and 25 years, in the United States has increased by approximately 160% since 2013 [1,2]. While most young adults who experience suicidal thoughts do not go on to make a suicide attempt, suicidal thoughts are indicators of risk that warrant attention and intervention [3]. Among adults who experienced mental health symptoms in the last year, young adults have the lowest rates of formal mental health care treatment [2]. Structural (eg, cost, accessibility, and provider availability) and attitudinal (eg, stigma, strong preferences for self-management) barriers are particularly important for determining help-seeking behavior [4] and contribute to low rates of mental health treatment engagement [4-6].

Current approaches to suicide prevention underserve young adults facing structural or attitudinal barriers to mental health services. Most prevention efforts are delivered in the context of mental health interventions via formal outpatient, inpatient, emergency department, and other clinical care settings. Prevention is also delivered in informal settings such as crisis helplines, targeting individuals before suicidal crises arise (eg, in schools or workplaces) in an effort to bring suicide prevention approaches to those who need it in places they are most likely to access it [7]. As most young adults with suicide-related thoughts and behaviors do not engage in formal mental health treatment, where most evidence-based suicide prevention is delivered [8-15], and they experience strong preferences for self-management, young adults are less likely to encounter evidence-based suicide prevention interventions.

The high need for preventive interventions and low formal mental health treatment engagement among young adults experiencing suicidal thoughts and behaviors [1,2] signal a critical gap in suicide prevention infrastructure. Digital suicide prevention interventions are an important avenue by which access to evidence-based suicide prevention care can be increased. Digital suicide prevention interventions offer the benefit of being able to circumvent many of the attitudinal and structural barriers that prevent young adults from accessing evidence-based preventive interventions [4-6]. They can be accessed on young adults’ devices (eg, smartphones) and used privately.

The safety planning intervention (SPI) [16], a brief intervention aimed at interrupting the progression from suicidal ideation to suicidal behavior by increasing coping ability, has typically been administered collaboratively by clinicians in clinical care settings across ages [17-19] and is considered standard of care [20,21]. The SPI typically includes 6 areas of focus: warning signs, coping skills, people for distraction, people for help, professional organizations and resources, and environmental safety [16]. The product of the SPI is often a brief document with personalized lists of resources and coping activities for each of the 6 focus areas. When delivered with fidelity, clinicians ask patients targeted questions and follow-up on patients’ responses to elicit highly personalized information that can enhance the efficacy of an individual’s safety plan by making it as relevant as possible to the individual’s life circumstances [22]. However, safety planning is frequently delivered by individuals facing barriers to implementation including insufficient training and time [23,24], which poses a risk to safety plan quality and efficacy.

The SPI is one of the most common digital suicide prevention intervention elements [25]. There is an emerging literature on automated digital SPIs that guide users through the creation of a highly personalized safety plan [25]. Several have been shown to be acceptable [26-29] and even demonstrate that resultant safety plan quality can be comparable to collaboratively developed safety plans [30,31]. Extant literature on digital SPIs primarily focuses on broad adult samples and narrow adolescent populations [25]. Boudreaux et al [26] piloted an automated, computer-based SPI that enabled adults who experienced suicide-related thoughts and behaviors to complete a safety plan and offered brief guidance on how to complete the safety plan. The resultant safety plan was a 1-page PDF that users could download, access on different devices, and share with trusted others. Melvin et al [27] and Rainbow et al [29] developed a mobile app–based approach called BeyondNow that includes a customizable SPI with prepopulated suggestions for each of the 6 safety planning steps and offers the ability to edit or share the completed safety plan and a quick access button to a crisis helpline. The app is publicly accessible and has strong acceptability metrics and is associated with promising improvements in suicide-related coping. Finally, Methi et al [28] created an automated web-based e-SPI specifically designed for adolescents that includes age-appropriate instructions, examples, and illustrations that assist users in completing the online safety plan via text-fill boxes. The content adolescents add is converted to an online safety card that users can save. These efforts highlight that automated safety planning is feasible to implement and that there are opportunities to engage individuals in new ways, expanding beyond single-session safety planning and crafting safety plans via mediums that are more conversational in nature.

Among this emerging literature, several key needs and preferences have surfaced. First, having quick access to information such as one’s documented safety plan, crisis resources, or personal contact information can be extremely valuable during a crisis as crisis states make it difficult to navigate complex user interfaces [32,33]. Second, there is a favorable view of digital artifacts (eg, photos, songs) that inspire hope and increase motivation for living [27]. Third, across the literature, users of digital SPIs have ambivalence about whether or how they want to involve others in their personal networks in the creation or use of their safety plans [32,33]. One way early digital SPIs have addressed this issue is through the use of optional prescripted messages users can generate when outside of crisis states and send to trusted others to elicit social or instrumental support [32]. Fourth, extant literature highlights that strong privacy practices emerge as critical priorities for users of digital safety plans [33,34]. Fifth, the ability to download and edit created safety plans after their initial creation is critical for ensuring safety plans remain relevant and accessible over time [27,34,35]. Finally, using validating language that minimizes assumptions about a user (eg, that they have a network of others that can provide support during a crisis) and ensuring the digital SPI can provide examples or menus of items if a user has difficulty generating safety planning content independently can prove beneficial [35].

The vast majority of digital SPIs are mobile app or browser based [25,36], meaning that individuals must visit a specific app or website in order to interact with or create their safety plan. In this study, we aimed to understand the potential of an SMS text messaging–based safety plan creation approach. Texting has the technological affordance of being a conversational medium, potentially addressing some of the shortcomings of automated digital safety plans that are documentation only. By eliciting information relevant to safety planning in a conversational manner, an automated texting–based SPI may be able to increase the personalization and relevance of digital safety plans. SMS text messaging is the most commonly used communication medium and young adults are the age group sending the greatest number of SMS text messages [37,38]. It is more widely used than social media apps [39]. Therefore, an SMS text messaging–based SPI is capable of meeting young adults in a space where they are already spending a significant amount of time. Indeed, prior work has shown that texting interventions for suicide prevention have been acceptable to users [40-45].

The relatively sparse existing literature on the needs and preferences of digital SPI users is focused on the app- or web-based designs. Further, it lacks focus on the needs of the adult age group with the highest rates of suicide-related thoughts and behaviors, but the lowest rates of treatment-seeking: young adults. Moreover, there is a paucity of literature regarding automated, interactive, texting interventions to support suicide prevention, broadly, and safety planning, more specifically.

### Objective

In this study, we aimed to understand the needs, preferences, constraints, and design considerations for an SMS text message–based automated safety planning tool for young adults who have lived experience of suicide-related thoughts and behaviors. Informed by prior literature, our work was guided by the following research questions: (1) What are the broad needs and preferences of young adults who experience suicide-related thoughts and behaviors for suicide prevention? (2) What are the features that can support young adults with suicide-related thoughts and behaviors to create an effective safety plan?

## Methods

### Participants

A total of 30 participants between the ages of 18 and 24 years were enrolled in this study (Table 1). Individuals were eligible to participate if they resided in the United States, were between the ages of 18 and 24 years, owned a smartphone, were proficient in English, and endorsed past 2-week suicidal ideation. Suicidal ideation was assessed using a modified item from the DSM-5 (Diagnostic and Statistical Manual of Mental Disorders, Fifth Edition) Cross-Cutting Symptom Inventory (DSM-5-CCSI) [46], “During the past 2 weeks, how often have you been bothered by thoughts of actually hurting yourself [or ending your life]?” Disambiguation of suicidal thoughts from nonsuicidal self-injurious thoughts was assessed via the Depression Severity Index—Suicidality Subscale (DSI-SS) [47], a 4-item self-report measure assessing the frequency and intensity of suicidal thinking. Participants were not eligible to participate if they endorsed symptoms consistent with mania or psychosis in the last 2 weeks (assessed via the DSM-5-CCSI) or the presence of strong suicidal desire assessed via item 9 on the Beck Depression Inventory—Second Edition [48] and a subsequent score of 2 or 3 (range 0-3).

**Table 1:** Participant characteristics.

Characteristics			Values
Age, mean (SD)			21.1 (1.9)
**Gender, n (%)**			
	Male	3 (10)	
	Female	22 (73)	
	Nonbinary	5 (17)	
**Transgender, n (%)**			4 (13)
**Race, n (%)**			
	Black/African American	3 (10)	
	American Indian/Alaska Native	1 (3)	
	Asian	5 (17)	
	Native Hawaiian/Pacific Islander	0 (0)	
	White	18 (60)	
	More than one race	2 (7)	
	Prefer not to answer	1 (3)	
Hispanic/Latino, n (%)			3 (10)
Total DSI-SS^a^ score (4 items, with each coded between 0 and 3; range 0-12), mean (SD)			4.8 (1.6)
Ever seen a therapist or mental health professional, n (%)			25 (83)
Currently seeing a therapist or mental health professional, n (%)			11 (37)
Plan to see a therapist or mental health professional in the next 8 weeks, n (%)			17 (57)
Never seen a therapist or mental health professional and does not plan to see one in the next 8 weeks, n (%)			1 (3)

^a^DSI-SS: Depression Severity Index—Suicidality Subscale.

### Procedures

Individuals were recruited via an advertisement embedded on Mental Health America’s (MHA) website. The advertisement was shown to individuals who visited a resource page about suicidal thoughts and behaviors, endorsed suicidal or self-harm ideation in the past 2 weeks on MHA’s online screening website, or endorsed past-month suicidal ideation on MHA’s nonsuicidal self-injury survey. Individuals who clicked the ad were directed to a Northwestern University–hosted informational web page about the study. Interested individuals could elect to provide informed consent for eligibility screening via Northwestern University’s instance of REDCap [49,50] and were subsequently routed to the eligibility screening surveys.

If eligible, interested individuals could review the REDCap-based study consent documentation and the code of conduct for engaging with research staff and fellow participants on the private research forum. The code of conduct detailed guidelines such as refraining from posting identifying information or detailed descriptions of self-harm, suicide methods, or behaviors. The code of conduct also overviewed harassment and moderation policies. To be enrolled in the study, individuals needed to provide affirmative informed consent and agree to abide by the code of conduct.

Participants engaged in 1 of 2 asynchronous remote community (ARC) [51-53] groups—akin to an online focus group. We used ARC to increase the engagement of underrepresented populations by decreasing barriers related to synchronous or in-laboratory research tasks [54]. Additionally, the group format was chosen to facilitate conversation between adults with shared experiences. ARC methods are used in the field of human-computer interaction to facilitate conversation, elicit several perspectives simultaneously, and provide the environment for participants to build on each other’s creative contributions [51]. ARC groups were held between November and December 2022, or February and March 2023. Two ARC groups were held to increase the sample size and diversity of responses to several lines of questioning and protect against group effects as well as to gather data on lines of inquiry that were not covered in the first group. ARC groups involved participants registering for a private online forum using FocusGroupIt [55] in which researchers and participants could create posts visible for all in the study and with which participants could engage or respond. Each group comprised 15 participants. Past work demonstrates similar sample sizes are sufficient to elicit diverse perspectives [56]. Participants were provided with automatically generated anonymous usernames (eg, participant 1, participant 2). Each ARC group consisted of 8 total prompts, 1 every 3 days. Prompts focused on terminology young adults used for suicidal ideation, prompting events for suicide-related thoughts and behaviors, safety plan use, and technology to support both safety planning and help-seeking.

Research staff monitored forums daily for posts violating the posted code of conduct. Offending posts were deleted, and the original poster was contacted by research staff with an explanation of why the post was removed, and, if needed, to conduct a risk assessment. Only 1 such post was removed and there was no indication of suicide-risk elevation for the poster.

### Data Analysis

The corpus for this study included 3 prompts from each group containing relevant data to answering the primary research questions (see Multimedia Appendix 1). These prompts comprising the corpus covered perceptions of safety planning via SMS text messages (group 1, prompt 4; group 2, prompt 3), the role of automated messages in supporting safety planning (group 1, prompt 5; group 2, prompt 4), use of a safety plan after it is created (group 1, prompt 7), and the role of crisis services in suicide prevention care (group 2, prompt 7).

Forum transcripts were analyzed using Thematic Analysis [57,58] to extract key needs, preferences, and design considerations for a digital safety planning tool. Thematic analysis was facilitated using Dedoose software (Version 9.0.9) [59]. One coder (JM) read through the corpus and generated an initial codebook via open coding responses to each of the 3 prompts from each ARC group. Coding occurred at the sentence level within a participant’s post. Two additional coders (SAP and TL) read through the entire corpus to familiarize themselves with the data and then applied the initial codebook to overlapping transcripts. All 3 coders then met to revise the initial codebook. Discrepancies were discussed until a consensus was reached among all coders. Importantly, the analysis software, Dedoose, facilitates the attribution of code applications to the research staff who coded the data, enabling the ability to identify and resolve coding discrepancies. All coders then divided the remaining data and applied the revised codebook so that the entire corpus was coded by at least two coders. Coders met 3 more times to discuss and resolve discrepancies. The saturation of codes was determined to be reached once all discrepancies in codes were resolved and no additional codes were identified in the overlapping transcripts. Coders then met to organize codes into axial themes iteratively.

### Positionality Statement

At the time of this study, JM was a clinical psychologist and faculty member with a background in both clinical and research work in suicide prevention. He held funded grants focused on increasing access to care through the design, development, and evaluation of digital mental health interventions. SAP was a research assistant who supported the administration of the ARC methods described in this paper. Both JM and SAP interacted with participants whose data comprise the study corpus through study procedures such as email and, in some cases, interviews. To maximize objectivity during coding and analysis, all participants were assigned a coded ID number. TL was a research assistant external to the project and had no prior contact with any study participants.

### Ethical Considerations

Before beginning any screening or study procedures, all interested individuals and participants in this study provided affirmative informed consent, both for initial eligibility screening and subsequently for participation in the study. Participants were compensated up to US $80 for responding to the study prompts and for commenting on other participants’ posts.

Throughout the study, participants’ data were, and continue to be, considered confidential and protected. Only research personnel and individuals securing appropriate credentials and data use agreements were able to access study data and complete transcripts. Study transcripts were deidentified and maintained on secure encrypted servers at Northwestern University.

All study procedures were administered at Northwestern University and all study procedures were approved by the Northwestern University Institutional Review Board (project number STU00217191).

## Results

### Young Adults’ Perspectives on Automated Digital SPIs: Treatment Mechanisms, Core Elements, and Skill-Building Features

Across participants in our ARC groups, young adults expressed their need for automated digital SPIs. Young adults discussed (1) the treatment mechanisms an automated digital SPI must address, (2) core elements of the technology that affect the perceived usefulness and acceptability of an automated digital SPI, and (3) key skill-building features they would like to see in such an intervention.

### Meeting Young Adults’ Needs

#### Key Needs for SPI Support: Self-Management and Social Connection

We identified 2 key needs that young adults thought an SPI should support. The first need was for self-management and the second was for social connection.

Empowering Young Adults to Manage Their Own Symptoms by Supporting Coping Skill Acquisition and Use

Young adults wanted an automated digital SPI to support the self-management of their suicide-related thoughts and behaviors through coping skills that foster a sense of self-empowerment. Self-empowerment approaches enable young adults to rely on their own skillfulness to effectively cope with their suicide-related thoughts and behaviors, and to avoid the stigma they associated with help-seeking. Some participants already possessed coping skills that facilitated self-empowerment and shared how these coping skills allowed them to navigate future suicidal crises:

I have never thought about using one of the crisis services, as I never felt as though I have needed to use one. I think it would be a bit embarrassing for me, as I strongly dislike talking about depression and/or suicide with others. I have also always been able to talk myself down without the need for a crisis service intervention.

G2-P2

G2-P2’s statement suggests that through practice in talking themselves down, they have been able to mitigate escalating suicidal crises and this experience provides confidence in their own ability. While participants, in general, noted the potential value of a tool to support coping skill use and acquisition, others such as G2-P3 highlighted the potential benefits of an approach that involved other people:

I ultimately chose not to use these [crisis helpline] services because I was able to eventually re-ground myself, however, in retrospect, I think it would have been more beneficial to talk to someone about this in the moment rather than going through it alone.

Young adult participants described the impact they believed an automated SMS text messaging–based safety planning tool could have on their self-empowerment in moments of need. G1-P4 shared that by creating a safety plan with the assistance of a digital tool, an individual is taking the lead in planning for their own coping. They described that one of the “benefits would be reassurance of the feeling of not being alone from something you created.” G1-P15 also highlighted the benefits of a self-created safety plan, commenting, “the fact that the individual will be able to change it, and have the power over their safety would probably make a lot of people who use the plan a lot more secure.” Overall, young adult participants were clear that an automated safety planning service would need to be able to provide them with the ability to choose how they cope with their symptoms.

Young adult participants shared that one of the motivations for using self-management tools was prior experiences of invalidation when discussing their suicide-related thoughts and behaviors with others. As G2-P12 recalled,

After reaching out to a hotline, I had to speak with a campus therapist and she laughed at me for considering suicide because my problems seemed “frivolous” or “overexaggerated”. I haven't been able to trust a hotline since, and after that one incident, I've cut myself off to dozens of resources that may help me with the fear they may joke about me in the same manner.

This experience of stigma, wherein the participant expected help and was instead met with judgment-worsened symptoms sowed distrust in systems and made future help-seeking less appealing, ultimately leading to decreased support and increased social isolation. As a result, young adults noted that it was important to have resources that enabled them to self-manage their symptoms and that were offered in a welcoming and nonjudgmental tone, mitigating the need to disclose suicide-related thoughts and behaviors to others to obtain support. For example, G1-P13 shared how messages could support effective coping, but would need to strike the right tone “I think automated messages like this would be extremely helpful in providing a structured way to create a safety plan. [...] these messages should be welcoming, even if they aren't actually run by a person, and include greetings or transitions.”

Participants described how an automated digital SPI delivered via SMS text messaging could foster self-empowerment by providing actionable coping resources in an easily accessible format. G2-P14 shared, “[the intervention] would need to be automated, where it texts my questions and I respond and it can provide me with the appropriate part of my personalized safety plan.” G2-P14’s comment depicts a scenario in which a user of an automated safety planning service is asked targeted questions that elicit personalized coping resources and then these personalized coping resources are sent back to the user at opportune times, facilitating self-management of suicidal crises and self-efficacy by enabling an individual to use coping resources effectively. This approach was echoed by others, for example, G1-P8 noted “Having the automated messages send me reminders for self-care and other things based on what I write in response to the messages (plus what I already have in my safety plan) would be great as well.”

Some participants shared how a digital SMS text messaging–based approach must accommodate change over time to fully support self-management of suicide-related thoughts and behaviors. As an individual’s circumstances, resources, and skillfulness change over time, what works for them at the outset will need to be updated to be more relevant and effective. G1-P6 shared that, “So much can change in just a second, and what worked for me yesterday might not work for me today.” Editing and maintaining one’s safety plan was seen as a critical component of self-empowerment in that it enables a constant cycle of being able to evaluate a safety plan’s efficacy against real-world suicide-related thought and behavior and crisis mitigation and management attempts. The ability to edit and maintain a safety plan allows for modification and adaptation in the event of actual or anticipated underperformance so that it can perform better in the future.

#### Drawing on Social Connections

The second core element many young adults in our study wanted was an automated digital SPI to support self-management of suicide-related thoughts and behaviors by supporting users in connecting with others. Participants had different ideas about how this could be accomplished, but the vast majority agreed that interpersonal connections were critical for any suicide-related thought and behavior intervention. The findings that follow highlight how components that support interpersonal connection may be critical targets in an automated intervention aimed at addressing suicide-related thoughts and behaviors, and could potentially work by creating accountability structures, creating openness to connection with others, fostering a feeling of being cared for, eliciting outside perspectives, and recruiting help when needed.

Creating social accountability was a core function participants said was important to consider for the self-management of suicide-related thoughts and behaviors. Participants noted that it was difficult to commit to using certain coping skills when in a suicidal crisis, but accountability to others would make it more likely for them to use the planned coping skills. One participant highlighted the utility of others to maintain accountability structures:

I do feel like a lot of safety plans are made with that individual having to keep themselves accountable in mind. But, part of a safety plan can include other people/ resources that can help in those times and in some ways keep you accountable and safe.

G2-P16

As G2-P16 articulates, the system could encourage the engagement of trusted others in the digital intervention to enhance the efficacy of digital safety planning.

Participants also wanted a digital intervention to facilitate connections with trusted others in their network. G2-P7 put it clearly, “I need human interaction more than anything right now.” This participant’s perspective highlights that many young adults are aware of the need for connection, especially in the context of crisis states, and that it is necessary to, first, build and, second, reach out to those support networks when needed. G2-P3 stated, “when experiencing feelings of loneliness/isolation alongside suicidal thoughts, it is especially beneficial to have social support mechanisms in place.” Young adults were open to an automated digital SPI but emphasized that it needed to also facilitate connection and strengthen ties to trusted others.

Participants imagined that an automated intervention could connect individuals to their support networks and allow young adults to get outside perspectives from mental health professionals or trusted others. When experiencing suicidal thoughts, young adults shared that it is challenging to access perspectives outside of their own, but acknowledged that it can be helpful for diverting or avoiding an escalating suicidal crisis. As a result, participants raised the importance of obtaining outside perspectives:

Sometimes it is really hard to notice signs of crisis in yourself, and having something point it out to you could be really helpful. I’ve noticed in myself that sometimes when I get really worked up I don’t notice how dangerous my behavior has become until a loved one points it out to me and expressed their concerns. It’s helpful to get an outside perspective.

G1-P6

While many participants wanted other people involved in the perspective-taking process, some did not. G2-P7 shared how experiences with past treatment-seeking cast doubt on the utility of involving others to offer outside perspectives, “The best therapy, in my opinion, is taking time to yourself and getting to the root of the issue alone. It was the best thing I could have ever done for myself.” However, participants discussed that an automated system should nonetheless prompt an individual to consider different ways of looking at one’s circumstances, experiences, and values providing an outside perspective that could serve a similar function without integrating a human in the loop by default. As G2-P3 states, “these messages could serve as gentle reminders of values and the things that are important to people, [...] I would be more likely to respond to these messages if they contained thought-provoking questions that probed my values.”

There was a minority of participants who shared that a potential drawback to an automated SMS text messaging–based SPI is that it had the potential to maintain or exacerbate social isolation by reducing a point of potential human contact such as calling or texting with a crisis counselor. For example, G2-P2 shared, “even if it designed for you, the digital aspect of the text is a bit isolating.” An SMS text messaging suicide-related thought and behavior intervention may be acceptable for many, but participants cautioned that if the interpersonal considerations were not sufficiently designed for, this type of intervention could cause harm. Specifically noting that automated suicide prevention interventions could be misused in place of a point of human contact (eg, crisis counselor support), potentially worsening subjective experiences of isolation.

### Technology Needs

#### Technological Needs for Automated Digital SPIs: Transparency, Privacy, and Usability

The young adult participants in our study also discussed needs related to the technological aspects of an automated digital SPI. Specifically, they highlighted the importance of (1) transparency or clarity regarding expectations, assumptions, and limitations of automated digital SPI; (2) privacy or the practices, policies, and technologies that limit exposure of personal information; and (3) usability, convenience, and accessibility of the intervention.

#### The Need for Transparency

Participants detailed the important ways in which being transparent in the context of an intervention for suicide-related thoughts and behaviors is critical to uptake and efficacy. One participant shared how transparency about crisis helpline practices helped them feel more comfortable using a particular crisis helpline:

Even just having some ideas of what may be asked of my or what to expect, vocabulary to express my feelings, etc. may help make the experience of asking for help much less scary and more accessible.

G2-P13

G2-P13 highlighted how, in the context of crisis helplines, having information about the scripts and procedures counselors follow before making the call can help put callers at ease by facilitating mental preparation. This in turn can reduce uncertainty during a crisis call, reduce fear about disclosing one’s suicide-related thoughts and behaviors to a stranger, and increase how readily accessible a helpline is to a prospective caller.

Similarly, in the context of an automated SMS text messaging–based SPI, young adult participants described that information about how the program functioned (eg, how many messages the system sent) and guidelines on the limits of confidentiality would be important when considering whether to use such a service. As G1-P3 articulated, “I have really bad anxiety so I am immediately thinking of well what if I do/say something “wrong” will they have a list of numbers and contact people and tell them.” Given the potential real-life consequences of mandated reporting laws, even if relatively rare (eg, involuntary hospitalization, removal from one’s home, or physical harm from emergency responders), participants in our study wanted transparency about the kind of information that would result in the automated intervention activating a mandated report. As G1-P3 alludes to, knowing in advance how the system responds to user input and operationalizes mandated reporting can help ameliorate symptoms of anxiety while being instructive as to how to get the most benefit from the service.

#### The Role of Strong Privacy Practices

In addition to transparent information about how a suicide-related thought and behavior prevention intervention functions, the underlying assumptions it makes about users, and the procedures to which it adheres, young adult participants also expressed the importance of privacy including (1) autonomy over data and data use, (2) anonymity, and (3) discretion.

Participants wanted to ensure they maintained control over their data. In the context of an SMS text messaging–based SPI, participants were concerned that their messages could be read by another individual, the information they share could be reported to third parties, and that the messages they sent would be identifiable. As G2-P13 shared, “I would be concerned about privacy though. I would want to know that what I shared over text would not be read by another person without my consent, and that what I shared would not be traced back to me or reported to someone.” By contrast, others saw the ability to create a safety plan via SMS text messages as a way to maintain control over how, when, and what the safety plan contained which they viewed as a form of data autonomy.

Another important technical specification for suicide-related thought and behavior-focused interventions was the ability to remain anonymous. As G2-P9 noted, “there is a fear of being traced and how it is not completely anonymous because the phone number is linked to you.” Anonymity was one of the factors that influenced whether or not participants would use a crisis service. For example, despite one participant’s misinterpretation of the National Suicide and Crisis Lifeline’s processes, they noted that a key factor in making the Lifeline an attractive intervention was the ability to remain anonymous (The National Suicide and Crisis Lifeline maintains confidentiality except in cases of imminent risk and they do not enable completely anonymous contacts. Their Information Technology infrastructure and counselors collect callers’ and texters’ personally identifiable information such as phone numbers, IP addresses, or volunteered other information that may be linked back to an individual.).

Relatedly, participants wanted digital or remote suicide-related thought and behavior-related interventions to be discreet. This was especially true when considering the use of a texting–based SPI. Participants were particularly concerned that people in their lives with physical access to their devices, but no or partial knowledge of their suicide-related thoughts and behaviors, might be able to access material that would result in an unwanted disclosure of an individual’s suicide-related thoughts or behaviors. G2-P5 noted, “I relate to the fear of being found out. Even with the promise of anonymity I’m still worried about someone in my life finding out and reporting me.” Another participant, G2-P12, contextualized this well-represented fear around a digital intervention, “I definitely would also be nervous someone could see it on accident, because that's definitely happened to me before where I opened my phone and people I was eating lunch with saw what I had recently seen.” While participants expressed general concerns around access to SMS text message data (eg, G1-P3 “I think the privacy is a big concern.”, G1-P2 “I think many people feel vulnerable about their mental state and want it treated with as much privacy as possible.”), most of the specific concerns about discretion and privacy focused on people in one’s day-to-day environment gaining access to information and how that disclosure would negatively affect an individual’s relationships. As G1-P6 explains, “most texting apps on phones don’t have locks or passwords as far as I know, and if someone got a hold of the person’s phone they would have immediate access to intimate and private details about the texter’s mental health.”

#### Usability, Convenience, and Accessibility

Participants shared a number of technical considerations related to an automated digital SPI. Among them was the importance of having a highly functional user interface. Participants were already familiar with the texting medium but noted that text-based menu navigation, relative to graphical user interfaces, could become tedious and frustrating. For example, G1-P8 said, “Text messages have a very specific, not customizable format. For something like a safety plan, I'd much prefer an app or website where I could work through my plan on a page or multiple pages, however that would look and be designed.” However, participants largely agreed that texting–based safety planning could offer an important level of convenience and accessibility that may be especially important for self-management of suicide-related thoughts and behaviors. For example, G2-P9 noted, “The benefits is that it is easily accessible though and your phone is everywhere with you so if you need to refer back to it you will have it with you.”

Participants also noted that an SMS text messaging–based service can make safety planning accessible to individuals that experience anxiety speaking to others about their concerns, in ways that synchronous voice call support cannot. G1-P1 shared, “I think the idea of texting gives me much less anxiety than having to call.” Therefore, specific communication channels may provide different levels of comfort, based on participants’ associations and past experiences.

Participants preferred tools that were efficient to use and did not require significant time or effort. This was especially true for participants who imagined using an automated digital SPI while in crisis. Further, if a program requires significant effort on the part of the individual, it could be demotivating and unhelpful. As G1-P13 shared, “I would be more likely to respond to these messages if they didn't take a lot of effort to respond to--if they involved a long, inconvenient chain of messages, for example, I wouldn't feel motivated to respond.” It is critical that messages are focused and direct, but that they also have some semblance of personality. Participants like G1-P11 shared how they wanted messages styled, “The messaging dynamic should be concise but not totally devoid of feeling, even if it is totally automated.” Participants wanted messages that made interacting with a tool interesting without being overly demanding.

### Skill Building Features

Beyond intervention and technological requirements, participants in our study shared numerous preferences and feature suggestions for an automated digital SPI that helped support skill building, not simply skill documentation—extending the role and function of a safety plan. For example, participants shared that they wanted self-tracking features so that they could identify patterns in their suicide-related thoughts and behaviors and build insights over time. G1-P4 shared one way this feature could be integrated into the larger automated digital SPI:

it could work in conjunction with tools where you can mark your feelings and behaviors, and once you know how you feel prior to an episode, it could help identify when you may potentially be feeling depressed or experiencing suicidal thoughts or behaviors.

Others wanted suggestions for coping skills. This augments traditional safety planning by making automated digital SPIs a coping idea generation and refinement system, hosting a repository for useful coping skills that might spark inspiration for a user, especially if that user is having difficulty generating coping skills on their own. One participant shared how this might work:

I think that using specific questions to personalize someone's safety plan could be really useful. One big problem I have with the general concept of safety plans is that I feel that I wouldn't know what specific things to add to it, this would definitely solve that issue. I think specificity would definitely help me answer or respond to those messages, I feel less motivated to answer messages from automated services where the prompt is super vague or not very engaging. Other text messages that would be helpful to receive could be resources to distract or help cope with difficult situations. Another way the service could ask for things to incorporate into the plan without directly asking could be to ask for the user's interests or things that might help them calm down.

G1-P11

Relatedly, some participants wanted reminders to use coping skills. While safety plans have traditionally been a static document, the translation to an automated digital SPI enables the system to prompt the user to assess the need to use coping skills through periodic reminders. As G1-P2 shared, “I personally feel like getting reminders to relax and check in with myself would be helpful, since suicidal thoughts can be brought on by stress.”

Taken together, participants shared that through reminders, skill suggestions, and self-tracking features, an automated digital SPI presents an opportunity to build skillfulness and insight for a potential user, rather than simply documenting effective coping skills.

## Discussion

### Principal Findings and Design Implications

This study aimed to understand the needs, preferences, and design requirements and features necessary for an automated SMS text messaging–based suicide prevention intervention. Our results suggest that young adults who experience suicide-related thoughts and behaviors are open to embedding suicide prevention interventions within SMS text messages, but these interventions must be designed to meet their unique needs and preferences.

One of the important needs arising from our ARC groups was the importance of social connection and accountability in automated digital SPI design. Young adults who experienced suicide-related thoughts and behaviors were interested in interventions that strengthen existing protective factors and social connections. This finding aligns with the Interpersonal Psychological Theory of Suicide [60,61] and the Three-Step Theory of Suicide [62,63], which each identify social disconnection as a key driver of suicidal desire and ideation. An automated digital SPI, such as the one proposed in this study, may offer psychoeducation to users on methods of leveraging existing social connections to build social accountability for symptom self-management. It may prompt users to consider available coping activities that foster a sense of connection (eg, letter writing, watching a movie with others). Additionally, an automated digital SPI may be able to support users in crafting messages to trusted others to solicit support around safety plan use and offer resources that users can send to trusted others to educate them on how to respond to someone experiencing suicidal thoughts (eg, tips to mitigate unhelpful reactions and methods of engaging in nonjudgmental listening). Importantly, our results also show that connection to trusted individuals within participants’ personal networks was particularly valued relative to simulated connection or companionship achieved exclusively through texting with an automated chatbot. Designers of automated SPIs should consider designs that encourage and support real-world interpersonal connections with people in users’ personal networks; otherwise, they could unintentionally exacerbate a sense of isolation.

Our finding that young adults believed that having social accountability may support their use of a digital safety plan, as well as their application of coping skills, also sits within the context of digital health literature that suggests, for some, framework-based human support and accountability [64,65] can facilitate longitudinal engagement in digital interventions [66]. Although human support aimed at engaging users in a digital health intervention is conceptually distinct from the social accountability young adults saw as valuable in facilitating the use of a safety plan—not necessarily a digital health intervention—there is conceptual overlap that signals the importance of others for facilitating the use of a therapeutic tool that may, at times, be difficult to engage with.

Participants in our study wanted to be highly informed about how an automated digital SPI would handle personal data. This finding has implications for the design of digital suicide prevention interventions broadly. Intervention systems must proactively communicate meta-information about how the intervention operates, as this has the potential to increase confidence and comfort using the intervention. Prior work demonstrates that individuals are more likely to make use of crisis helpline resources when they are provided alongside barrier-reduction interventions that target informational gaps such as misunderstandings of crisis helpline practices with regard to active rescues (ie, emergency responders being called to make contact with an individual) before receiving a referral to crisis helplines [67]. Moreover, U.S. Suicide and Crisis helpline (ie, 988) data suggest that several of the top barriers to contacting the Suicide and Crisis helpline are due to fears about law enforcement being called and about privacy and confidentiality [68]. These top concerns are addressable through robust transparency practices that frontload information about the decision rules and processes used within the intervention and by its administrators. For instance, having clear and simple language before a user engages in the intervention that addresses (1) how and when confidentiality is broken and emergency services are called; (2) how data inputs are used such as contact information for individuals a user can reach out to for help during a crisis; (3) if, when, and how trusted others are contacted, and (4) proactive psychoeducational information about how privacy and confidentiality are maintained within the program (ie, data security measures). Finally, it is important to offer resources that empower users to ask questions about privacy, confidentiality, and clinical decision-making in other contexts such as formal treatment settings and crisis helpline calls. Thus, while our findings suggest the value of self-management to reduce the need for crisis services, they also suggest important ways that crisis services can improve.

Themes raised across our ARC groups highlight the importance of ensuring that an automated SPI is usable and convenient. In the context of SMS text messaging–based safety planning, this means the system design must incorporate bidirectional or interactive messaging. To date, SMS text messaging interventions for suicide prevention [40-44] have primarily used unidirectional messaging (originating from the intervention system and being sent to the user), leaving individuals with limited opportunity to engage with intervention material in ways that reflect their lived experiences. To ensure an SMS text messaging–based automated SPI is convenient and usable, the system must use the conversational medium to be interactive. Eliciting safety plan entries by prompting users to consider new or different aspects of coping and prompting users to be highly specific are all unique affordances offered by texting. To make an SMS text messaging system usable and convenient, a system must also leverage a text-based navigation menu. This enables users to access settings, jump to different parts of their safety plan, and modify elements of a safety plan, if needed. To this end, participants highlighted that while texting may be useful for safety plan creation, having multiple methods of editing a safety plan may be necessary if the navigation menu becomes cumbersome. Thus, offering multiple modes of interacting with one’s safety plan (ie, via SMS text messages and via a web-based editing tool) may be required to make an automated digital SPI maximally usable.

Our results reveal an overlap between young adults’ stated needs and preferences and innovations in the interactive text–based market. Our results highlighted that young adults had several needs and preferences that may lend themselves to innovation using emerging technologies. Specifically, young adults expressed a desire for a digital SPI service to support, among other needs, (1) coping skill acquisition and use, (2) quick and simple menu navigation and easy access to desired information, (3) interpersonal connection to trusted others, and (4) clear privacy policies. The recent commercial availability of highly customizable large language models (LLMs), a class of machine learning model trained on massive linguistic data sets and designed to process and generate text-based data, has increasingly been integrated into text-based mental health interventions in the form of chatbots, content generators, and as classifier algorithms that code or categorize data so that an intervention becomes more responsive to user inputs [69]. LLMs integrated into digital chatbots can tailor responses based on context, previous interactions, and user inputs. This emerging technology is beginning to be integrated into digital suicide prevention tools, especially as it relates to the detection of suicide-related user-generated content [70], and comes with significant risks in addition to substantial promise [71]. While our study did not explicitly seek to understand the role of LLMs in an automated digital SPI, participants in our study offered important insights that are relevant to intervention development in the context of a market where commercial LLMs are available and actively being incorporated into different digital mental health technologies.

First, as it relates to supporting coping skill acquisition and use, LLM-based messages may be launched to support users in ideating coping skills when they have difficulty generating their own. Moreover, the ability of LLMs to personalize content based on individuals’ contexts and inputs creates opportunities to increase the relevance of a user’s safety plan at the moment. With a user’s existing safety plan as context, LLMs may be able to support idea generation about how to overcome barriers to coping (eg, if an individual’s safety plan contains going for a walk outside as a coping skill, but weather is prohibitive, LLM-based messages may be able to support a user identifying relevant solutions, such as going for a walk indoors, taking protective gear, using other coping skills on one’s safety plan). Second, LLMs may be able to improve the experience of navigating rigid text-based menus embedded in an SMS text messaging intervention by enabling the system to accept user responses that do not exactly match preprogrammed keywords that direct a user down different branching logic trees and instead allowing users to respond using natural language. Third, young adults were clear that they wanted a digital SPI to support interpersonal connections to other humans. While they were open to an automated digital SPI, connection to real people was crucial. LLM-based chatbots have often been used as conversational agents [70], some of which have personified the conversational agent. This may exacerbate a suicide-specific vulnerability: isolation and loneliness. Past work highlights that young adults with depression were concerned that using a robot for companionship could worsen their symptoms by making the absence of real human companions more apparent [72]. More research is needed to understand whether LLM-based suicide prevention tools should present themselves as personified agents or as self-service tools. Finally, given participants’ emphasis on the importance of privacy and transparency, if an LLM was to be integrated into an automated digital SPI to enhance messages, it would need to (1) proactively make transparent ways in which user inputs would be used to train models and (2) disclose the potential risk of confidentiality breaches through the LLM itself, as has been documented in other contexts [73].

By engaging young adults with lived experience of suicide-related thoughts and behaviors to understand their needs, preferences, constraints, and the context of existing technological solutions, we can identify design requirements that are able to be applied across digital suicide prevention tools.

### Limitations and Future Directions

This study is limited in several important ways. First, while we engaged in rigorous user-centered design practices to understand the needs and preferences of young people who experience suicide-related thoughts and behaviors, we were only able to engage a relatively small number of individuals, and our results may not be generalizable to broader young adult populations. In our sample of 30 participants, 11 (37%) were in formal mental health treatment, and 17 (57%) planned to seek formal treatment in the coming months. These treatment-engaged numbers are greater than the approximately 12% of US adults who have experienced suicidal thoughts and sought treatment in the past year [2]. Moreover, we recruited exclusively from MHA’s website which may inherently limit the perspectives of individuals included in the study to those who are open to technology-assisted interventions and services, and potentially more actively engaged in self-management of mental health symptoms. While we aimed to cover a broad range of topics in our ARC groups, our discussions were guided by researcher-posted prompts and centered on eliciting needs and preferences as they related to safety planning or texting. To this end, in this study we did not systematically examine needs and preferences for risk management in self-guided digital safety planning tools; however, future work must aim to better understand the needs and preferences across interested parties (eg, consumers, research participants, clinicians, organizations) as it relates to risk management procedures. Finally, individuals at the highest levels of suicide risk (ie, individuals who may have had elevated levels of suicidal desire and intent) were not eligible to participate in this study and thus their voices were systematically excluded as the study format lacked appropriate support for these individuals. Future work should focus on applying these design considerations and assessing their value within the context of deployable digital suicide prevention interventions.

### Conclusion

Engaging young adults in the design process of a digital suicide prevention tool revealed critical considerations that must be addressed if the tool is to effectively expand access to evidence-based care to reach young people at risk for suicide-related thoughts and behaviors. Specifically, automated digital SPIs must support building skillfulness to cope effectively with suicidal crises, deepening interpersonal connections, system transparency, and data privacy.

## Appendix

Multimedia Appendix 1 Initial research prompts used to generate threaded conversations during asynchronous remote community online focus groups.

## Abbreviations

ARC: asynchronous remote community
CCSI: Cross-Cutting Symptom Inventory
DSI-SS: Depression Severity Index—Suicidality Subscale
DSM-5: Diagnostic and Statistical Manual of Mental Disorders, Fifth Edition
LLM: large language model
MHA: Mental Health America
SPI: safety planning intervention

## Data Availability

Because of the sensitive nature of text-based data and the potential risk of reidentification, data are not publicly available. However, deidentified data are available upon reasonable request with the appropriate data use agreements in place.
