# Supplementary material for: Automated Digital Safety Planning Interventions for Young Adults: Qualitative Study Using Online Co-design Methods
Source: JMIR Form Res. 2025 Feb 26;9:e69602. doi: 10.2196/69602 (PMC11904377; doi:10.2196/69602)
Supplement: Multimedia Appendix 1 [file formative_v9i1e69602_app1.docx]

**Appendix 1**

**Initial Research Prompts Used to Generate Threaded Conversations During Asynchronous Remote Community Online Focus Groups**

| **Group and Prompt Number** | **Prompt Text** |
| --- | --- |
| Groups 1, Prompt 4 & Group 2, Prompt 3 | One way to use technology to help people navigate suicidal crises could be to send text messages. We’d like to know more about your feelings about text messaging. Specifically, imagine that you signed up for a program where you receive automated text messages that helped you create and use a safety plan on your phone.  What are your reactions to the idea of creating, editing, or reviewing a private and highly personalized safety plan via text message? What are some of the reasons for this initial impression?  Are there benefits or drawbacks of a safety planning tool that is delivered over text messaging? |
| Group 1, Prompt 5 & Group 2, Prompt 4 | One way text messaging could be applied to safety planning is to help people write down the things that work for them to “turn down the volume” on their suicidal thoughts. For example, someone may watch a Disney movie to cope with their suicidal thoughts, while another might play a video game to distract themselves from their own thoughts. An automated text messaging program might ask questions like, “What sorts of activities can you do to help take your mind off of problems?” The system would then include the person’s response as part of their safety plan.  How would you feel about getting automated messages like this while you create your safety plan? Would some messages be more helpful than others in helping you create a safety plan?  What would make you more or less likely to respond to these messages?  Are there other messages you would want to receive, or other ways a text messaging program could ask you for this information? |
| Group 1, Prompt 7 | In this session, we are interested in the ways you might interact with a safety plan after you have created it. This plan could be accessed in a few ways. For example, after you create the plan, the system could send you an image or an email with the filled-out plan (e.g., filling in your coping strategies, people you can ask for help, etc.). You could also scroll through your text messages to see the full conversation you had with the messaging program. What sort of record would you want to receive from the messaging program after you create your plan? Would it be helpful if you were able to edit your plan after creating it? If so, how would you want to do this? |
| Group 2, Prompt 7 | There are a number of crisis services that are designed to help individuals when they are experiencing suicidal thoughts or behaviors, as well as other general stressors. These include the National Suicide Prevention Lifeline, the Veterans Crisis Line, the Crisis Text Line, the Trevor Project, the Trans Lifeline, warmlines (e.g., where peers who have lived experience of mental health difficulties like experiencing suicidal thoughts listen and provide support), and many others. Each of these services are staffed by different folks and offer different services. Some serve broad populations, and others serve narrow ones like LGBTQ+ young people, some have online chat or text messaging services, others offer talking to a counselor on the phone, some are staffed by peers with lived experience of suicidal thoughts and behaviors, others may not be. Some do not call emergency services under any circumstances, and others only refer folks to emergency services in very narrow cases.  If you’ve ever thought about using one of the above crisis resources (or similar crisis services), what were the most important things about the service that made you consider using it?  Were there specific things that made you want to use one crisis service over another? Why or why not?  If you have ever thought about using one of these services, but ultimately chose not to, what stopped you or why did you end up not using it? |

:
